# Bladder paraganglioma, gastrointestinal stromal tumor, and SDHB germline mutation in a patient with Carney-Stratakis syndrome: A case report and literature review

**DOI:** 10.3389/fonc.2022.1030092

**Published:** 2022-10-28

**Authors:** Yihang Shi, Li Ding, Chengqiang Mo, Yanji Luo, Shaoqing Huang, Shirong Cai, Yanzhe Xia, Xinhua Zhang

**Affiliations:** ^1^ Department of Gastrointestinal Surgery, The First Affiliated Hospital of Sun Yat-sen University, Guangzhou, China; ^2^ Department of Pathology, The First Affiliated Hospital of Sun Yat-sen University, Guangzhou, China; ^3^ Department of Pharmacy, The First Affiliated Hospital of Sun Yat-sen University, Guangzhou, China; ^4^ Department of Urology Surgery, The First Affiliated Hospital of Sun Yat-sen University, Guangzhou, China; ^5^ Department of Radiology, The First Affiliated Hospital of Sun Yat-sen University, Guangzhou, China

**Keywords:** Carney-Stratakis syndrome, paraganglioma, gastrointestinal stromal tumor, succinate dehydrogenase deficient, germline mutation

## Abstract

**Background:**

Carney-Stratakis syndrome (CSS) is a rare dyad of paraganglioma (PGL)/pheochromocytoma (PHEO) and gastrointestinal stromal tumor (GIST). PGLs are neuroendocrine tumors of neural crest origin which are mostly found in the head, neck, and retroperitoneal space. GISTs are the most common mesenchymal tumors of the digestive tract, usually caused by *KIT/PDGFRA* mutations. Here, we reported a case of CSS with unusual bladder PGL and succinate dehydrogenase (SDH) deficient GIST due to a germline mutation in SDH-subunit B (SDHB) gene.

**Case presentation:**

A 39-year-old female patient initially diagnosed with gastric GIST and isolated pelvic metastasis was eventually found to be CSS with bladder PGL and SDH-deficient GIST after surgery. This patient underwent resection of gastric and bladder tumors, and postoperative pathology confirmed the diagnosis of CSS. According to the next-generation sequencing (NGS), the patient carried a germline mutation in the SDHB gene, which was the cause of the disorder. The patient had no tumor recurrence with regular follow-up in 10 months.

**Conclusions:**

CSS is an autosomal genetic disorder with no gender difference in incidence, and PGLs are more frequent than GISTs. SDH germline mutation is the molecular biological mechanism of CSS while the most common type is SDHB mutation. The unique mechanism of tumorigenesis including hypoxia and hypermethylation caused by SDH deficiency renders target therapy with tyrosine kinase inhibitors ineffective, therefore complete surgical resection is the optimal treatment in the absence of tumor metastases.

## 1 Introduction

Carney-Stratakis syndrome (CSS) is a rare dyad inherited in an autosomal incomplete dominant manner that includes paraganglioma (PGL)/pheochromocytoma (PHEO) and gastrointestinal stromal tumor (GIST). It was distinguished from the Carney triad in 2002 by Carney et al. through a description of 12 patients from 5 unrelated families (1). Carney triad included pulmonary chondromas in addition to GIST and PGL (2). Afterward, adrenocortical adenoma and esophageal leiomyoma were added as components of the triad (3). Germline mutations in genes encoding mitochondrial succinate dehydrogenase (SDH) complex including SDH-B, SDH-C, and SDH-D in CSS patients were identified by McWhinney and Pasini in 2007 (4). The dysfunction of the SDH complex was noted to cause the development of PGL/PHEO and GIST in CSS (5).

PGL and PHEO are neuroendocrine tumors of neural crest origin while 30%-40% of them are familial (6). The vast majority of PGL and PHEO are multiple tumors. PHEO occurs in the adrenal medulla; those originating in the extra-adrenal sensory or parasympathetic ganglia are called PGL. These PGLs closely related to the autonomic nervous system ganglia can be divided into sympathetic and parasympathetic, the former can show symptoms of increased catecholamine secretion, and the latter are usually nonfunctioning. Paraganglioma can be found anywhere in the body, from the skull to the pelvic floor, most commonly in the neck and retroperitoneal space (7).

GIST is the most common mesenchymal tumor of the digestive tract, with approximately 10 to 15 cases per million population (8, 9). Since Hirota et al. revealed *KIT* gene mutations in GIST in 1998 (10), approximately 85% of GIST patients are now considered to be due to *KIT/PDGFRA* gene mutations (11). In *KIT/PDGFRA* wild-type GISTs, approximately 50% of patients are negative for SDHB by immunohistochemistry (11), termed SDH-deficient GISTs, which have several special clinical manifestations, such as earlier age of onset, multiple lesions, most of them originating in the stomach, and more prone to lymph node metastasis (12, 13). SDH-deficient GIST can also be part of some clinical syndromes, such as the Carney triad and CSS.

Here, we report a case of CSS with gastric GIST synchronized with an unusual site of PGL from the bladder, and review the relevant literature to better understand the clinical and histopathological features, genetic pathogenesis, and molecular features of CSS.

## 2 Case presentation

### 2.1 Preoperative examination

A 39-year-old woman came to our hospital for the gastric and pelvic masses incidentally found during a routine body examination in the local hospital for two weeks in October 2021. The enhanced Computed Tomography (CT) of the abdomen and pelvis revealed two masses located in the fundus of stomach and the right side of the pelvis, respectively, which were considered to be GIST originating in the stomach with solitary pelvic metastases by the local hospital.

**Figure 1:**
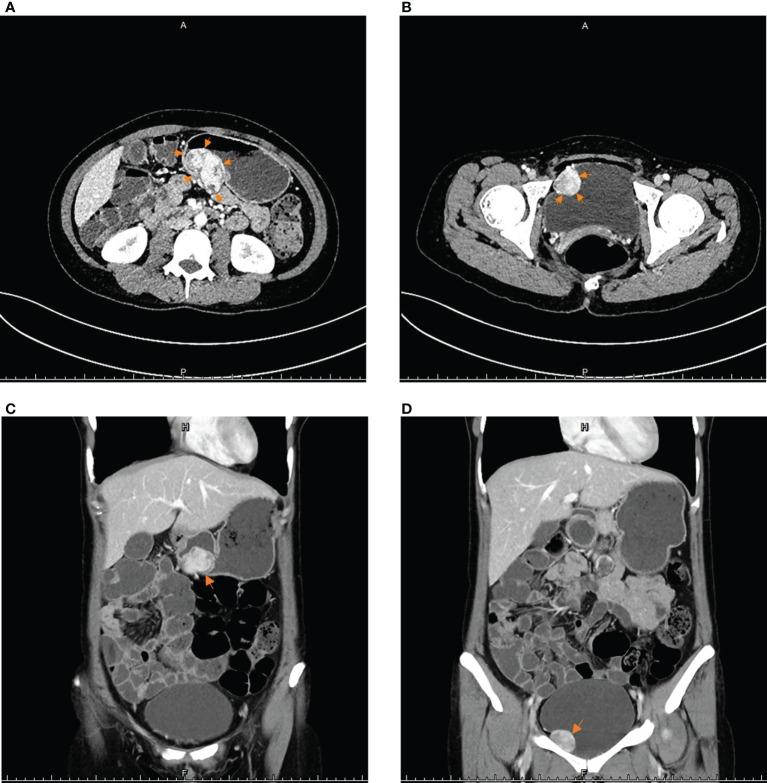
The imaging results of contrast-enhanced CT scans of the abdomen and pelvis: **(A, C)** The arrows point to a soft tissue mass approximately 33 mm×54 mm in the posterior wall of the gastric body. **(B, D)** The arrows point to a protrusion into the right wall of bladder, approximately 26 mm×29 mm in size of round lumps.

The patient then came to our hospital for treatment. Her physical examination was normal, no mass was palpated in her abdomen, and her blood pressure and heart rate in a calm state were 109/70 mmHg and 72 bpm, respectively. The re-examination of enhanced CT of the abdomen and pelvis showed a soft tissue mass of approximately 33 mm×54 mm in the gastric wall on the greater curvature of the gastric body with clear boundaries and a smooth surface. A nodule of 26 mm × 29 mm was seen on the right anterior side of the pelvic wall, which was unclearly demarcated from the bladder wall. And there were no obvious abnormal signs in the rest of the body (**Figures 1A-D**). Combined with imaging findings, these two lesions were more likely to be considered as primary tumors from different sources other than gastric GIST with peritoneal metastasis.

### 2.2 Surgical treatment

**Figure 2:**
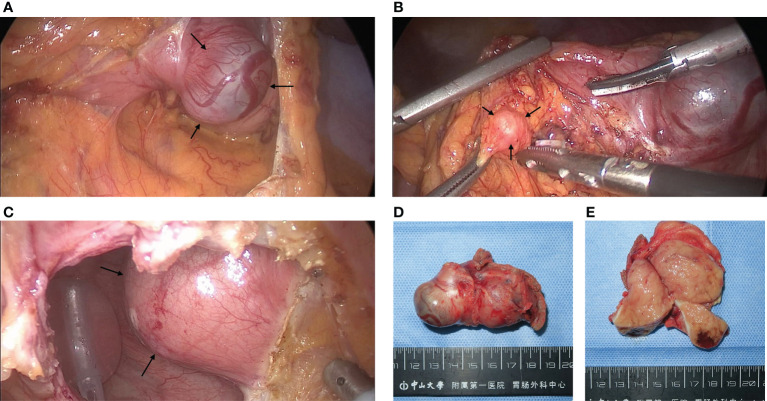
Pictures of GIST and PGL during and after surgery: **(A)** Intraoperative finding of a stromal tumor in the posterior wall of the gastric corpus which the arrows point to. **(B)** An enlarged nodule adjacent to the GIST pointed by arrows, which was initially suspected to be a lymph node metastasis from the stromal tumor but was diagnosed as hyperplastic fibrous connective tissue by cytopathology. **(C)** After incision of the peritoneum and the anterior wall of the bladder, a mass pointed by arrows on the right side of the bladder protruding into the bladder was found, which was confirmed by pathology as a PGL. **(D, E)** GIST after resection and its transverse section, approximately 6 cm in length. The tumor capsule was intact and limited, the section was brown, located in the inherent muscle layer.

After the MDT discussion, the patient underwent laparoscopic surgery on October 9 in 2021, During the operation, a mass of approximately 55 mm × 35 mm in the posterior wall of the gastric body was found, and wedge resection of stomach was performed (**Figures 2A, D, E**). At the same time, several nodules suspected of enlarged lymph nodes on the greater omentum adjacent to the mass were excised together (**Figure 2B**) and sent for frozen-section. The pathology revealed fibrous connective tissue. When dividing the mass from the bladder, the patient’s blood pressure rapidly rose to 180/120 mmHg. The anesthesiologist resolutely suggested that the operation be interrupt, and the arterial continuous blood pressure monitoring was performed before resuming the operation. According to the above situation, an ectopic PHEO was suspected, the urologist was invited for intraoperative consultation, and confirmed that the mass came from the right side of the bladder apex (**Figure 2C**). Complete excision of the mass and bladder repair were performed.

### 2.3 Postoperative diagnosis and follow-up

#### 2.3.1 Results of pathology and next-generation sequencing

**Figure 3:**
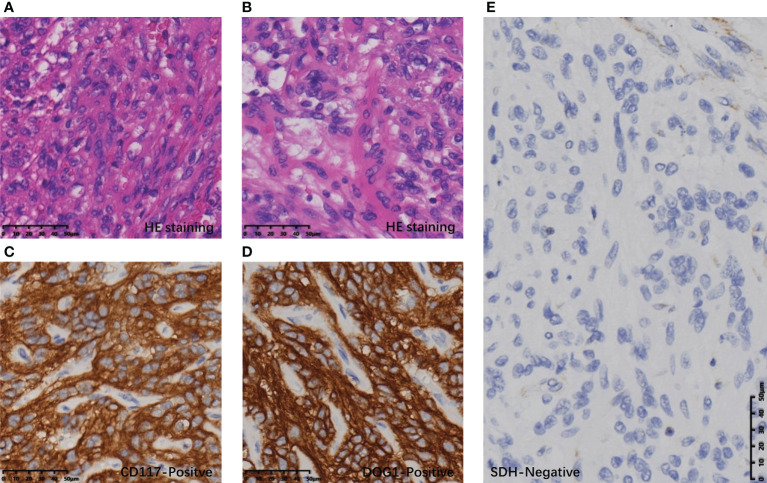
Histopathological and immunohistochemical results of GIST: **(A)** Spindle-shaped tumor cells on HE staining. **(B)** Epithelioid tumor cells on HE staining. **(C)** Tumor cells with diffuse CD117 positivity. **(D)** Tumor cells were diffusely positive for DOG1. **(E)** Tumor cells were diffusely SDH negative under the SDH positive control of vascular endothelial cells.

**Figure 4:**
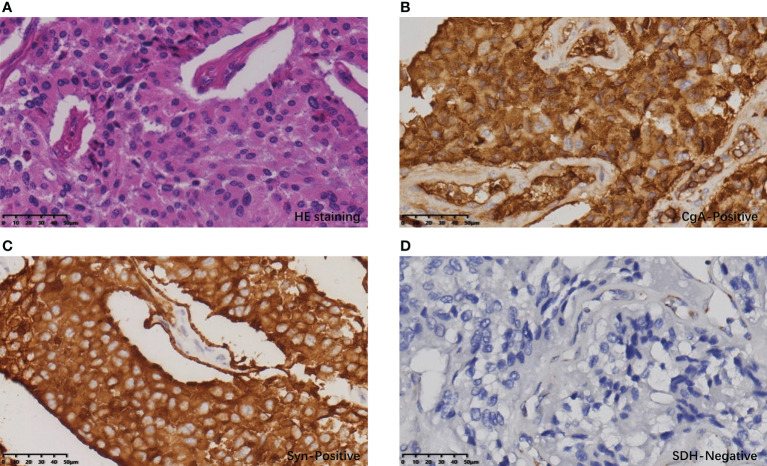
Histopathological and immunohistochemical results of PGL: **(A)** The cells of the bladder tumor were arranged in nests under HE staining, with abundant cytoplasm and stroma rich in thin-walled blood vessels. **(B)** Tumor cells showed diffuse CgA positivity. **(C)** Tumor cells showed diffuse Syn positivity. **(D)** Tumor cells showed diffuse SDH negativity; vascular endothelial cells served as an SDH positive control.

Postoperative pathology confirmed that the gastric mass was an SDH-deficient GIST, which was composed of epithelioid cells and spindle cells under HE staining, with moderate atypia. The mitotic count was 2 per 5 mm^2^. The immunohistochemical (IHC) result showed tumor cells were CD117(+), DOG-1(+), SDHB(-), and Ki-67 5% (**Figure 3**). The rest of the specimens also included 15 lymph nodes, and no tumor cell metastasis was found. The bladder mass was a PGL. Under HE staining, it was composed of tumor cells of uniform size with abundant cytoplasm arranged in nests, with IHC CgA (+), Syn (+), SDHB(-), and Ki-67 2% (**Figure 4**). The results of next-generation gene sequencing revealed that the patient had a SDHB c.137G>A germline mutation. According to the above results, she was diagnosed with Carney-Stratakis syndrome of bladder PGL and gastric GIST, caused by SDHB germline mutation.

#### 2.3.2 Follow-up

The patient recovered well after surgery and was discharged on the 6^th^ postoperative day. During the follow-up, the patient told us that the symptoms including dizziness, palpitations, and sweating which happened during urinating disappeared completely after removal of the tumors. Because of the germline mutation of the SDHB gene, we advised her first-class family members to undergo next-generation gene sequencing, but they refused. The patient was receiving regular clinical, and imaging follow-ups. The patient remained recurrence-free up to this report being prepared at 10 months after surgery.

## 3 Discussion

In a study published in 2002, Carney and Stratakis identified an autosomal dominant association between PGL and GIST by investigating 12 patients (7 males and 5 females) from 5 different families, and they defined this inherited, incompletely penetrant syndrome as CSS (1). PGLs are neuroendocrine tumors that originate in the neural crest, and approximately 30% to 35% of them are hereditary. Hereditary PGLs can be components of many clinical syndromes, such as multiple endocrine neoplasia 2, von Hippel-Lindau (VHL) disease, type 1 neurofibromatosis, Carney triad, and CSS (7). Several genes, including RET, VHL, NF1, and SDH, are thought to be involved in hereditary PGL. GIST, the most common mesenchymal tumor of the gastrointestinal tract, is thought to originate from Cajal cells in the gastrointestinal tract. Approximately 85% of GISTs occur due to *KIT/PDGFRA* mutations. GISTs with no *KIT/PDGFR* mutation are referred to as wild-type. SDH-deficient GIST is a subtype of wild-type involved in the formation of CSS and the Carney triad. The SDH deficiency in tumor cells of GIST and PGL is thought to be associated with germline mutations, hypermethylation, or somatic mutations of SDH-encoding genes (14–16).

In this paper, we reported a typical case of CSS with bladder PGL and SDH-deficient GIST. A total of 39 case reports of Carney-Stratakis syndrome between 2002 and 2022 were retrieved from the PubMed database using the keywords “Carney-Stratakis syndrome” and “Carney- Stratakis”, with an average age of 25 years (9-59 years), including 21 male patients (53.8%) and 18 female patients (46.2%) (1, 5, 17–30). In the 39 cases, 37 (94.9%) patients developed PGL, 29 (74.4%) patients developed GIST, and 26 (66.7%) patients had concurrent PGL and GIST. GIST sites were mentioned in 21 cases, of which 20 (20/21, 95.2%) originated in the stomach. Nine (10/29, 34.5%) patients had GIST lymph node or distant metastasis, and four of them had GIST recurrence after primary tumor resection. Of the 37 patients with PGL, 21 (56.8%) had systemic multiple PGLs, with the most common sites including bilateral paracarotid, paraaortic, bilateral adrenal glands, and mediastinum. Twenty-two (56.4%) of all patients had a clear family history of related disorders. Genetic testing was performed in 19 (48.7%) patients, all of which were found to carry SDH gene germline mutation, including 10 (10/19, 52.6%) patients with SDHB mutation, 4 (4/19, 21.1%) patients with SDHC mutation, and 5 (5/19, 26.3%) patients with SDHD mutation. The GIST in this patient we reported has the characteristics of SDH deficiency type, such as primary gastric and SDH negative. In particular, the PGL of this patient with CSS was primary in the bladder and had typical symptoms of sympathetic excitation such as cold sweating, palpitations, headaches when urinating, which was different from previously reported cases of CSS. The patient’s genetic testing revealed that she had a germline mutation in the SDHB gene, which also proved the typicality of this CSS case.

### 3.1 Mechanisms underlying SDH mutations leading to tumorigenesis

SDH is a mitochondrial enzyme complex consisting of four subunits, SDHA, SDHB, SDHC, and SDHD, which are encoded by nuclear genes located at 5p15.22, 1p36.13, 1q23.3, and 11q23.1, respectively. It is involved in both the tricarboxylic acid cycle (catalyzing the oxidation of succinate to fumarate) and in the respiratory electron transfer chain (transferring electrons to coenzyme Q). The SDHA subunit is a flavoprotein that binds and catalyzes succinate to fumarate (31). SDHB is an iron-sulfur protein that together with SDHA constitutes the catalytic core and combines SDHA with SDHC and SDHD. SDHC and SDHD are responsible for anchoring the complex to the inner mitochondrial membrane (32). Subunits inactivation caused by mutations in either coding gene results in SDH dysfunction and negative SDHB immunohistochemical staining. This makes it possible to determine whether the expression of SDH is normal by SDHB immunohistochemical staining and to determine whether the gene encoding SDHx has been mutated (33). In 2007, McWhinney et al. described SDHB, SDHC, and SDHD germline mutations in patients with Carney-Stratakis syndrome (34). Later, studies by Passini et al. reconfirmed this finding and proposed that mitochondrial complex II dysfunction caused by mutations in the genes encoding the SDH subunit and loss of normal alleles may be responsible for PGL and GIST in Carney-Stratakis syndrome and their pathogenesis (5). Therefore, tyrosine kinase inhibitors, which make a great achievement in the field of targeted therapy, have no therapeutic effect on this type of GIST (12).

It is not completely clear that the dysfunction of SDH leads to tumors; several mechanisms have been proposed. One of them is the activation of the pseudohypoxia pathway (35). Due to SDH dysfunction, the tricarboxylic acid cycle can be hindered and succinate is accumulated, which inhibits propyl hydroxylases (PHDs) resulting in induction of the hypoxic response despite normoxic conditions (pseudohypoxia) (36). In normal, hypoxia inducible factor-1 (HIF-1) will be recognized and degraded by VHL protein after being hydroxylated. Accumulated succinate inhibits the propyl hydroxylases which can hydroxylate the HIF-1 in the cases that SDHx genes are mutated. Therefore, hydroxylation of HIF-1 is decreased, and they escape degradation. Subsequently, they translocate to the nucleus and activate genes that facilitate angiogenesis, cell proliferation, and glycolysis.

Based on a summary of reported Carney-Stratakis syndrome cases (**Table 1**), we found that germline mutations in the SDHB and SDHD genes were the most common. Miettinen et al. reported the same SDHB c.137G>A germline mutation as the case in this report (34).

**Table 1:** Review of the Carney-Stratakis syndrome cases.

Family/Patient	Sex	Age	PGL	GIST	Gene	Mutation	Reference
1/1	F	20	M, NF	NA	ND	ND	Carney et al.2002 (1)
1/2	M	16	M, NF	Gastric, M			
2/3	F	21	M, F	Gastric, M, MET			
2/4	F	14	M, F	NA			
3/5	M	46	M, F	Gastric, M, MET			
3/6	M	40	M	NA			
3/7	M	10	M, F	NA			
4/8	F	18	F	NA			
4/9	M	9	NF	Gastric, M			
5/10	M	23	M, F	NA			
5/11	F	31	NF	NA			
5/12	M	16	NA	Gastric, M, MET			
6/13	M	37	NA	ND	SDHB	c.72+1G>T	Passini et al.2008 (5)
7/14	M	12	ND	NA	SDHB	c423+1G>C	
7/15	M	12	NA	ND	SDHB	c423+1G>C	
8/16	F	26	M	ND	SDHB	c.45-46insCC	
9/17	F	29	ND	ND	SDHC	c.43C>T	
10/18	F	20	ND	ND	SDHC	c405+1G>A	
11/19	M	19	M	ND	SDHD	c.57delG	
4/20	M	9	M	ND	SDHB	large deletion	
12/21	F	20	M	NA	NA	NA	
12/22	M	15	M	ND	NA	NA	
13/23	M	19	M	ND	NA	NA	
14/24	F	38	F	Gastric, MET	SDHD	ND	Bailey et al.2020 (17)
15/25	F	45	NF	Gastric, M, MET	ND	ND	Recht et al.2020 (18)
16/26	M	39	M, F	Gastric	SDHD	ND	Nicholas et al.2015 (19)
17/27	M	59	M, NF	Gastric	NA	NA	Lecamwasam et al.2015 (20)
18/28	M	32	M.F	Gastric	SDHB	c166-170del	Jove et al.2014 (21)
19/29	M	24	M	Gastric	SDHD	c.14G>A	Tenorio et al.2012 (22)
20/30	F	12	M	Gastric, M	NA	NA	Vaughan et al.2011 (23)
21/31	F	29	F	NA	SDHB	ND	Bolland et al.2006 (24)
22/32	F	40	F	Gastric	SDHB	ND	Alrashdi et al.2010 (25)
23/33	M	33	M, F	Gastric	SDHD	ND	Ayala-Ramirez et al.2010 (26)
24/34	F	38	NF	Rectal, MET	SDHD	c.445_448dupATCT	Gasparotto et al.2016 (27)
25/35	F	22	NF	Gastric	ND	ND	Ghigna et al.2016 (28)
26/36	M	16	M, F	Gastric, MET	SDHC	Exon2 c.43 C>T	Stanley et al.2019 (29)
27/37	M	16	NF	Gastric, MET	SDHB	Exon1 c.24delC	Rinelli et al.2020 (30)
28/38	F	16	NF	Gastric, MET	SDHC	Intron3 c.287-1G>C	
29/39	F	34	NF	Gastric, MET	NA	NA	Fukada et al.2022 (37)

*M, multiple diseases; MET, metastasis; F, functioning; NF, nonfunctioning; NA, not available; ND, not detailed in article

### 3.2 Clinical and pathological features of PGL and GIST in CSS

PGL and GIST in CSS have unique clinical features due to their specific pathogenic mechanism. SDH-deficient PGL is usually multifocal and widely distributed. They may occur in the head and neck, back, mediastinum, retroperitoneum, adrenal gland, and pelvis, of which the head, neck, and retroperitoneum are the most common sites (7). Most PGLs that develop in the head and neck are nonfunctioning. Notably, the patient reported here had a solitary PGL on the right side of the bladder wall which did not show any symptoms when her bladder was relaxed. When urinating, the compression of the PGL by the detrusor muscle of the bladder might lead to the release of internally stored catecholamines into the blood, causing typical symptoms of sympathetic nerve activation including sweating and palpitations. She presented with a more than 20-year history of headache, pallor in appearance, cold sweating, and palpitations within 1 to 2 minutes after urination. The above symptoms only lasted for a few minutes and then relieved spontaneously which is similar to the previously reported clinical manifestations of bladder PGL (38, 39). Jove et al. reported a similar patient with CSS who had concurrent bladder and right retroperitoneal PGLs (21), and Lam et al. reported 2 cases of bladder PGL with voiding hypertension (40). It is suggested that although PGL in the bladder is rare, its specific clinical presentation can help doctors diagnose patients with similar conditions. The symptoms of the patient in this report had lasted for nearly 20 years, which suggested that her PGL might have initially developed 20 years ago. After 20 years of disease, the tumor did not metastasize or progress. Histopathologically, SDH-deficient PGLs typically present with nested tumor cells and prominent intrastromal vascular proliferation.

GIST is the most common mesenchymal tumor of the digestive tract, and its most common cause is abnormal activation of the tyrosine kinase pathway due to mutations in the KIT or PDGFRA gene. In addition, approximately 5% of GISTs are caused by loss of SDH function. They usually have SDH expression defects and are called SDH-deficient GISTs (16). Most SDH-deficient GISTs have mutations in the SDHx gene, and the most frequent mutation location is the SDHA subunit (16). However, in the CSS case reported here, no SDHA mutation was found. Some SDH-deficient GISTs have SDH dysfunction caused by hypermethylation of the DNA in the promoter region of the SDHC gene. No SDHx gene-related mutations have been detected in such GISTs, which are more common in patients with Carney triad (15). Most SDH-deficient GISTs originate in the stomach. In addition, the clinical features of SDH-deficient GISTs include multiple lesions, young age at onset, a prevalence in females, and a tendency for lymph node metastasis (16). However, according to a review of previous cases, CSS does not differ in incidence by sex (13). GISTs in CSS typically exhibit indolent biological behavior and are usually detected by routine physical examination. Based on a retrospective analysis of previously reported cases, we speculate that lymph node metastases in SDH-deficient GISTs may indicate a high risk of postoperative GIST recurrence. According to the current research results, adjuvant imatinib therapy has no obvious effect on SDH-deficient GISTs (41). Therefore, for patients with CSS with GIST lymph node metastasis, we recommend adopting a more active follow-up strategy to monitor the disease and administer timely treatment. Histopathologically, the most obvious feature of SDH-deficient GISTs is negative with SDHB immunohistochemical staining, which is the most used method for diagnosis. SDH-deficient GISTs are usually composed of interweaving epithelioid and spindle-like tumor cells, which is different from common GISTs.

### 3.3 Differentiation between CSS and Carney triad

Although both PGL and GIST in CSS have unique clinical features, and histopathological detection methods can be used as diagnostic aids, PGL and GIST are also part of the Carney triad. It makes the differential diagnosis between Carney triad and Carney-Stratakis syndrome difficult. Unlike Carney-Stratakis syndrome, due to the pathogenesis of germline mutations in the SDHx gene, no gene related to the pathogenesis of Carney triad has been found thus far. Carney once proposed that large segment loss of 1q was the most common genetic abnormality in Carney triad (42). Haller et al. found that patients with Carney triad had DNA hypermethylation of SDHC genes. These differences in genetic inheritance can help us differentiate between Carney triad and Carney-Stratakis syndrome (15). In addition, Carney triad is usually sporadic, while Carney-Stratakis syndrome is a familial genetic disorder, and positive family history can also be used as a basis for differentiation between the two (1).

### 3.4 Treatment of CSS

Surgical resection is the treatment of choice for both Carney-Stratakis syndrome and Carney triad. Once SDH-deficient GIST is diagnosed, surgery is the most appropriate treatment for the GIST and any related clinical syndromes. In addition, due to different biological behaviors, SDH-deficient GISTs are not suitable for the current evaluation system, such as the modified NIH evaluation criteria, in the evaluation of postoperative recurrence risk (43). Surgery is also recommended for PGL. In addition, genetic testing is warranted in patients with clinically suspected Carney-Stratakis syndrome or Carney triad, which can influence treatment options. For patients with germline mutations in the SDHx gene, it is also necessary to advise their immediate family members to undergo genetic counseling. For metastatic cases, traditional chemotherapy and radiotherapy have long been eliminated from the GIST treatment regimen, and imatinib is also not responsive (44). It is reported that multi-targeted TKIs such as sunitinib or regorafenib had certain effect on SDH deficient GISTs (45–47).

## 4 Conclusions

Carney-Stratakis syndrome is a dyad involving PGL and GIST, which is inherited in an autosomal incomplete dominant manner with no gender difference in incidence, and PGLs are more frequent than GISTs. Not every patient with CSS will develop both PGL and GIST. This case showed that the diagnosis of CSS should be considered in patients with SDH-deficient GIST with other solitary tumors. SDH germline mutation is the molecular biological mechanism of CSS while the most common type is SDHB mutation. PGLs in CSS are mostly multiple lesions, and the majority are in the neck and retroperitoneal space but may also be solitary in the bladder. The unique mechanism of tumorigenesis including hypoxia and hypermethylation caused by SDH deficiency renders target therapy with tyrosine kinase inhibitors ineffective, therefore complete surgical resection is the optimal treatment in the absence of tumor metastases.

## Author contributions

All authors contributed to the diagnosis and treatment of the patient. YS and LD drafted the work and wrote the manuscript. XZ and YX edited the manuscript, substantively revised it, and approved the re-submitted version. YX and SH provide substantial help to the writing of the article. CM and YL made substantial contributions to the treatment and diagnosis of the patient. All authors contributed to the article and approved the submitted version.

## Acknowledgments

We thank the patient and their families in this paper for their consent. Thanks to Ms. Huang Yanmei for the data collation work for this study.

## Conflict of interest

The authors declare that the research was conducted in the absence of any commercial or financial relationships that could be construed as a potential conflict of interest.

## Publisher’s note

All claims expressed in this article are solely those of the authors and do not necessarily represent those of their affiliated organizations, or those of the publisher, the editors and the reviewers. Any product that may be evaluated in this article, or claim that may be made by its manufacturer, is not guaranteed or endorsed by the publisher.

## Abbreviations

CSS, Carney-Stratakis syndrome; PGL, paraganglioma; PHEO, pheochromocytoma; GIST, gastrointestinal stromal tumor; SDH, succinate dehydrogenase; CT, Computed Tomography; PHD, propyl hydroxylases; HIF-1, hypoxia inducible factor-1; VHL, von Hippel-Lindau.
